# Colonoscopic perforation leading to a diagnosis of Ehlers Danlos syndrome type IV: a case report and review of the literature

**DOI:** 10.1186/1752-1947-5-229

**Published:** 2011-06-23

**Authors:** Mariam Rana, Omer Aziz, Sanjay Purkayastha, Josephine Lloyd, John Wolfe, Paul Ziprin

**Affiliations:** 1 Division of Surgery, Department of Surgery and Cancer, Imperial College London, St Mary's Hospital, South Wharf Road, London W2 1NY, UK; 2 Department of Histopathology, St Mary's Hospital, Imperial College Healthcare NHS Trust, London W2 1NY, UK; 3 St Mary's Hospital Regional Vascular Unit, Imperial College Healthcare NHS Trust, London W2 1NY, UK

## Abstract

**Introduction:**

Colonoscopic perforation is a rare but serious complication of colonoscopy. Factors known to increase the risk of perforation include colonic strictures, extensive diverticulosis, and friable tissues. We describe the case of a man who was found to have perforation of the sigmoid colon secondary to an undiagnosed connective tissue disorder (Ehlers-Danlos syndrome type IV) while undergoing surveillance for hereditary non-polyposis colorectal cancer.

**Case presentation:**

A 33-year-old Caucasian man presented to our hospital with an acute abdomen following a colonoscopy five days earlier as part of hereditary non-polyposis colorectal cancer screening. His medical history included bilateral clubfoot. His physical examination findings suggested left iliac fossa peritonitis. A computed tomographic scan revealed perforation of the sigmoid colon and incidentally a right common iliac artery aneurysm as well. Hartmann's procedure was performed during laparotomy. The patient recovered well post-operatively and was discharged. Reversal of the Hartmann's procedure was performed six months later. This procedure was challenging because of dense adhesions and friable bowel. The histology of bowel specimens from this surgery revealed thinning and fibrosis of the muscularis externa. The patient was subsequently noted to have transparency of truncal skin with easily visible vessels. An underlying collagen vascular disorder was suspected, and genetic testing revealed a mutation in the collagen type III, α1 (*COL3A1*) gene, which is consistent with a diagnosis of Ehlers-Danlos syndrome type IV.

**Conclusions:**

Ehlers-Danlos syndrome type IV, the vascular type, is a rare disorder caused by mutations in the *COL3A1 *gene on chromosome 2q31. It is characterized by translucent skin, clubfoot, and the potentially fatal complications of spontaneous large vessel rupture, although spontaneous uterine and colonic perforations have also been reported in the literature. The present case presentation describes the identification of Ehlers-Danlos syndrome type IV in a patient with a non-spontaneous colonic perforation secondary to an invasive investigation for another hereditary disorder pre-disposing him to colorectal cancer. Invasive procedures such as arteriograms and endoscopies are relatively contra-indicated in Ehlers-Danlos syndrome type IV. Alternatives with a lower risk of perforation, such as computed tomographic colonography, need to be considered for patients requiring ongoing colorectal cancer surveillance. Furthermore, management of vascular aneurysms in patients with Ehlers-Danlos syndrome type IV requires consideration of the risks of endovascular stenting, as opposed to open surgical intervention, because of tissue friability. Genetic and reproductive counseling should be offered to affected individuals and their families.

## Introduction

A colonoscopy may be performed for the investigation of symptomatic patients, as part of national colorectal cancer (CRC) screening programs, and for surveillance of high-risk patients. Colonoscopic perforation is a rare but serious complication of colonoscopy, with a reported incidence of 0.2% to 0.4% [1]. Factors known to increase the risk include transmural inflammation, colonic strictures, adhesions, extensive diverticulosis, and friable tissues, particularly tumors [1]. We describe a colonoscopic perforation leading to the unveiling of an occult connective tissue disorder in a man being screened for a strong family history of CRC.

## Case presentation

A 33-year-old Caucasian man was admitted to the Emergency Department of our hospital with acute abdomen and sepsis following a surveillance colonoscopy five days earlier at another hospital unit. He had previously been fit and well, although he had had two corrective surgeries for congenital talipes equinovarus in childhood and early adolescence. His family history was notable for CRC on the maternal side of his family. His mother and grandmother had died from colonic malignancy (his mother at age 42 years), and his cousin had been diagnosed with CRC at age 21 years. Furthermore, his maternal aunt had been diagnosed with endometrial cancer in her 40s and had recently been found to carry a mutation in the gene *hMSH2*, which is associated with hereditary non-polyposis colorectal cancer (HNPCC). As a result, he had now had five screening colonoscopies since the age of 21 years, although no abnormalities had been detected thus far.

**Figure 1:**
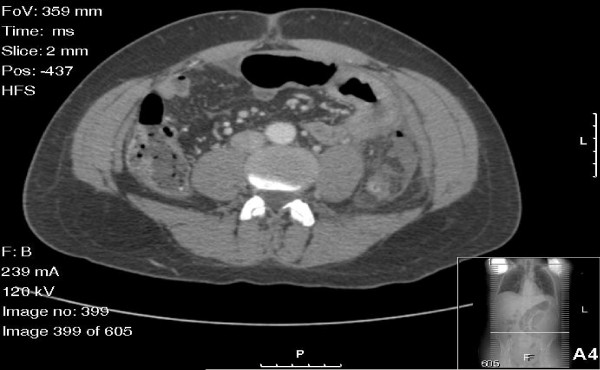
**Computed tomographic scan of the patient's abdomen showing pockets of free air and fluid in proximity to the jejunum and a thickened left colon with inflammatory stranding**. Inflammatory changes are present in the peritoneal fat, especially adjacent to the left colon. These findings are consistent with a perforated sigmoid colon.

**Figure 2:**
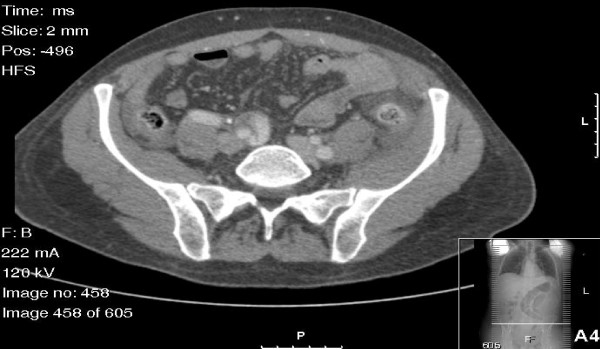
**Computed tomographic scan demonstrating the right common iliac artery aneurysm (2.7 cm in diameter) with dissection**. The left common iliac artery measures 1.4 cm.

His physical examination was notable for pyrexia and tachycardia with signs of localized left iliac fossa peritonitis. A computed tomography (CT) scan of the abdomen and pelvis showed inflammation of the sigmoid-descending colon junction and the adjacent loop of the proximal jejunum, which were associated with a small pocket of free air and free pelvic fluid (Figure 1). Incidentally, an isolated right common iliac artery (RCIA) aneurysm measuring 2.7 cm in diameter was also noted (Figure 2). As the clinical and radiological findings suggested a controlled localized perforation of the sigmoid colon, he was initially managed non-operatively with immediate bowel rest, intravenous fluid resuscitation and broad spectrum antibiotics. However, over the next 24 hours, he developed signs of more extensive peritonitis, so we proceeded to perform an emergency laparotomy.

Surgical exploration revealed generalized purulent peritoneal fluid and a small perforation of the proximal sigmoid colon. Colonic resection was performed with the formation of an end colostomy (Hartmann's procedure). The histology of the colonic segment was consistent with acute perforation with no identifiable cause and so was presumed to be due to his recent colonoscopy. The patient recovered well post-operatively and was discharged with outpatient follow-up by the Colorectal and Vascular Surgical units at our hospital.

**Figure 3:**
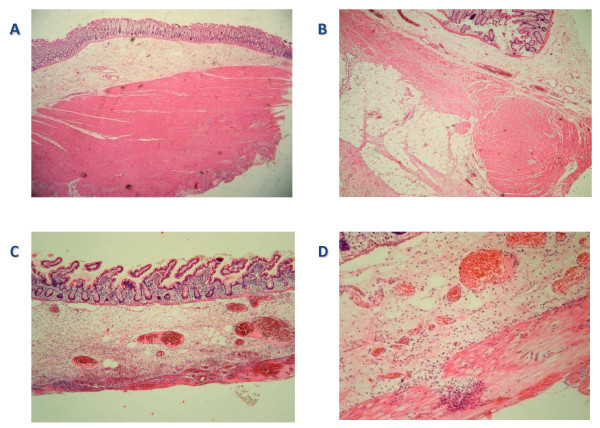
**Several microscopic histological slides obtained from the patient**. **(A) **A segment of normal colon from this patient. **(B) **A segment of abnormal colon and **(C) **a segment of abnormal small bowel showing patchy thinning and fibrosis of the muscularis externa. **(D) **Abnormal muscle with patchy fibrosis (sigmoid colon). It is also inflamed because of the perforation in the region.

Six months post-operatively, with no intervening complications, the patient had a reversal of the Hartmann's procedure. This was technically challenging because of dense fibrous adhesions and an apparently fragile small bowel, resulting in two small bowel resections and repairs of multiple enterotomies. Because of the complexity of the case, the colorectal anastomosis was covered by a defunctioning loop ileostomy. Histological analysis of the colonic and small-bowel specimens obtained during this surgery revealed patchy thinning and fibrosis of the muscularis externa, with complete absence of the layer in some bowel segments (Figure 3). There was no evidence of vasculitis or of a hollow visceral myopathic disorder. No amyloid was found, nor were there any features to suggest myositis. It was suggested that the focal defects in the muscle might be in keeping with a collagen vascular disease.

The patient was subsequently noted to have a high-arched palate, tethered earlobes, long fingers (although not hypermobile), and transparency (but not hyperelasticity) of the skin over the anterior chest with easily visible vessels. These clinical features, together with the history of congenital clubfoot, the operative and histological findings of bowel wall thinning, and the incidental finding of the RCIA aneurysm, were suggestive of an underlying collagen vascular disease, specifically Ehlers-Danlos syndrome type IV (EDS-IV), despite there being no family history of this disease. The patient was therefore referred for genetic testing.

A skin biopsy was performed to assess levels of type III collagen secreted by dermal fibroblasts (typically reduced in patients with EDS-IV [2]), although the results were inconclusive. Genetic testing was undertaken, and genomic sequencing revealed a heterozygous missense non-conservative mutation c.3256G > T in exon 45 of the collagen type III, α1 (*COL3A1*) gene, consistent with a diagnosis of EDS-IV. This mutation is predicted to replace the glycine at position 1086 of the triple-helix domain with a cysteine, and although it has not been previously reported in the literature, similar mutations within the triple helix are known to be associated with EDS-IV [3].

## Discussion

Colonic perforation is a rare but serious complication of colonoscopy, occurring most commonly in patients with diverticular disease or a strictured, severely diseased segment of colon and particularly affecting the sigmoid [4]. Friable tumors are also more liable to perforate [4]. This case illustrates the increased risk of endoscopic perforation in individuals with fragile tissues secondary to an underlying collagen vascular disease such as EDS-IV.

The exact prevalence of EDS is not known, but is estimated to be between one in 10,000 and one in 25,000 [5]. It comprises a group of hereditary connective tissue disorders which are differentiated into six main types according to the Villefranche classification [6,7]. The vascular type of Ehlers Danlos syndrome, EDS-IV, is a rare autosomal dominant disorder accounting for 5% to 10% of all cases of EDS [5]. It is caused by heterozygous germline mutations in the *COL3A1 *gene on chromosome 2q31 [2], which results in decreased extracellular type III collagen, leading to the loss of tensile strength of connective tissues, vascular structures, and hollow viscera. As a result, the condition is characterized by facial structural abnormalities, easy bruising, translucent skin, and clubfoot, as well as the potentially fatal complications of spontaneous rupture of large vessels and hollow organs, particularly the colon [8-10].

Hyperflexibility of the skin and joints in EDS-IV is less marked than they are in other types of EDS, so colonic perforation or aneurysm rupture may be the first presentation of the disease. As a result, EDS-IV in particular is associated with reduced life expectancy, with a median age of survival of 50 years [8]. Diagnosis of the disorder is made on the basis of clinical signs, as well as the demonstration that cultured dermal fibroblasts synthesize abnormal type III procollagen molecules, or by the identification of a *COL3A1 *gene mutation [8]. The *COL3A1 *gene mutation identified in our patient is a new mutation not previously reported in the literature, although similar mutations are well known to be associated with EDS-IV [3]. Specific mutations within the gene are not thought to accurately predict the extent or prognosis of EDS-IV [10], nor are they associated to the types of complications observed in this condition [8].

Approximately 80% of patients with EDS-IV have experienced at least one complication by the age of 40 years [8]. Bowel perforations tend to occur between the late teens and early 40s, with the sigmoid colon most often being affected [8]. As in the present case, the histology of colonic specimens in patients with EDS-IV typically shows changes in the caliber of the lamina muscularis and may demonstrate secondary diverticula formation due to reduced resistance of the submucosal soft tissue [7].

Previous reports have described spontaneous colonic perforations secondary to EDS-IV in the pediatric and adult patient populations [11,12], although the surgical management and outcomes of these patients have varied [12]. Some cases have been managed with subtotal or total colectomies, although most have been treated with colonic resection and formation of colostomy (Hartmann's procedure) [12]. Attempts at re-anastomosis following resection and diversion have been complicated by recurrent perforations and anastomotic leaks, presumably due to the bowel fragility, and have been compounded by adhesions as found in the present case. Consequently, some commentators have advised against restoration of colonic continuity and have advocated a subtotal colectomy as the first-line management of these patients [12].

The index case raises several interesting issues. First, many diagnoses of EDS-IV are retrospective and occur following a major complication. Thus, performing more extensive surgery prophylactically in the treatment of a localized colonic perforation may be an unlikely decision in the undiagnosed EDS patient, as in this case. However, if the condition is known or recognized, a total colectomy and ileorectal anastomosis need to be considered. In this case, these procedures would also have helped with regard to the patient's CRC risk.

Second, the present case illustrates the identification of EDS-IV in a patient with an atypical (non-spontaneous), presumably colonoscopy-induced perforation. Whereas large-vessel rupture in a young patient would normally prompt the clinician to consider an underlying connective tissue disorder, this case emphasizes the importance of considering EDS-IV in otherwise healthy young patients presenting with bowel perforation, a less well-known complication of the condition.

Given that there is no particular therapy to prevent further complications from occurring, however, the benefit of a retrospective diagnosis once such events have occurred is debatable. Nonetheless, knowledge of the disease may influence future surgical decisions, such as the extent of surgery in the treatment of further colonic perforations. Also, as in this case, the management of co-existent stable but expansive vascular aneurysms requires consideration of the increased risk of endovascular stenting because of tissue friability and the problems of open surgical intervention, particularly adhesions and suture tearing in the friable artery.

Third, in accordance with guidelines established by the International Collaborative Group on Hereditary Nonpolyposis Colorectal Cancer (ICG-HNPCC) [13], the index patient would continue to require biannual CRC screening on the basis of his family history. Colonoscopy is the current gold standard for surveillance of HNPCC families [13-15]; however, invasive procedures such as arteriograms and endoscopies are relatively contra-indicated in patients with EDS-IV [5]. Alternatives such as CT colonography, which carries a lower risk of perforation and has similar sensitivities for the detection of CRC [16], although less so for polyps measuring less than 10 mm, need to be considered for patients requiring ongoing surveillance for CRC. In the present case, however, the patient was later excluded as a carrier for the *hMSH2 *gene mutation identified in his aunt.

Finally, a diagnosis of this hereditary condition has implications for the patient's family, and genetic testing needs to be offered to living relatives. As the patient has a one in two chance of having an affected child, reproductive counseling as well as predictive, diagnostic, and prenatal testing should be made available.

## Conclusion

It is important to maintain a high index of suspicion of colonic perforation in post-colonoscopy patients presenting with acute abdomen. The sigmoid colon is the most frequent site of both colonoscopic perforations and spontaneous perforations occurring secondary to EDS-IV, possibly because of its anatomical characteristic of frequent redundancy. Colonic perforations in otherwise healthy young patients, together with suggestive history and examination features, should prompt the clinician to consider an underlying connective tissue disorder such as EDS-IV. This case illustrates the challenges of managing such patients.

## Consent

Written informed consent was obtained from the patient for publication of this case report and any accompanying images. A copy of the written consent is available for review by the Editor-in-Chief of this journal.

## Competing interests

The authors declare that they have no competing interests.

## Authors' contributions

MR was involved in the conception of the report, the literature review, and the preparation and submission of the manuscript. PZ reviewed and edited the case report. All authors were involved in the patient's care and contributed to the manuscript. All authors read and approved the final manuscript.
